# Reduction of Heavy Menstrual Bleeding in Women Not Designated as Responders to Elagolix Plus Add Back Therapy for Uterine Fibroids

**DOI:** 10.1089/jwh.2021.0152

**Published:** 2022-05-16

**Authors:** Elizabeth A. Stewart, David F. Archer, Charlotte D. Owens, Kurt T. Barnhart, Linda D. Bradley, Eve C. Feinberg, Veronica Gillispie-Bell, Anthony N. Imudia, Ran Liu, Jin Hee Kim, Ayman Al-Hendy

**Affiliations:** ^1^ Division of Reproductive Endocrinology, Department of Obstetrics & Gynecology and Surgery, Mayo Clinic and Mayo Clinic Alix School of Medicine, Rochester, Minnesota, USA.; ^2^ Department of Obstetrics and Gynecology, Eastern Virginia Medical School, Norfolk, Virginia, USA.; ^3^ AbbVie, Inc., North Chicago, Illinois, USA.; ^4^ Division of Reproductive Endocrinology and Infertility, University of Pennsylvania, Philadelphia, Pennsylvania, USA.; ^5^ Department of Obstetrics and Gynecology, Cleveland Clinic, Cleveland, Ohio, USA.; ^6^ Department of Obstetrics and Gynecology, Northwestern University School of Medicine, Chicago, Illinois, USA.; ^7^ Ochsner Health, New Orleans, Louisiana, USA.; ^8^ Department of Obstetrics and Gynecology, Morsani College of Medicine, University of South Florida, Tampa, Florida, USA.; ^9^ Department of Obstetrics and Gynecology, Columbia University, New York, New York, USA.; ^10^ Department of Obstetrics and Gynecology, University of Chicago, Chicago, Illinois, USA.

**Keywords:** elagolix, uterine fibroids, GnRH antagonist, heavy menstrual bleeding

## Abstract

**Objective:**

To assess outcomes of women with uterine fibroids (UFs) and heavy menstrual bleeding (HMB) treated with 300 mg elagolix twice daily plus add-back therapy (E2 1 mg/NETA 0.5 mg once daily) or placebo who were not considered responders in pooled analysis of two phase 3, 6-month randomized clinical trials (Elaris UF-1 and UF-2).

**Methods:**

Responders were defined as women who met both primary end point bleeding criteria (<80 mL menstrual blood loss [MBL] during the final month and ≥50% reduction in MBL from baseline to the final month) and either completed the study or discontinued due to predefined reasons. Thus, women termed nonresponders who were analyzed in this study who met neither or one bleeding end point or met both criteria but prematurely discontinued treatment because of adverse events, perceived lack of efficacy, or required surgical or interventional treatment for UFs were analyzed in this study. This *post hoc* analysis assessed mean changes from baseline in MBL, as well as adverse events.

**Results:**

Among 367 women receiving elagolix with add-back with observed data, 89 (24%) were not considered responders. Within this subset, 17 (19%) women met both bleeding criteria but prematurely discontinued treatment for the reasons mentioned above, while 23 (26%) met one bleeding criterion and 49 (55%) met neither bleeding criteria, regardless of discontinuation status. Among all nonresponders, a numerical trend toward greater mean reductions in MBL was observed in those receiving elagolix with add-back, compared with placebo group nonresponders. No differences in adverse events were observed between responders and nonresponders.

**Conclusion:**

Forty of 89 (45%) women with HMB and UFs who were classified as nonresponders in the UF-1 or UF-2 trials may have had a clinically meaningful response to elagolix with add-back therapy because they met at least one of the objective bleeding criteria. Clinical Trial Registration: Clinicaltrials.gov, NCT02654054 and NCT02691494. (NEJM 2020; 382:328–340) DOI: 10.1056/NEJMoa1904351

## Introduction

Uterine fibroids (UFs) are most common type of benign neoplasm, found in the myometrium of the uterus, and are associated with heavy menstrual bleeding (HMB).^1^ Elagolix, an oral gonadotropin-releasing hormone (GnRH) receptor antagonist, at a dose of 300 mg twice daily administered in combination with hormonal add-back therapy (estradiol 1 mg and norethindrone acetate 0.5 mg once daily) is currently the only FDA-approved oral treatment option specifically indicated for the management of HMB associated with UFs. While other nonsurgical treatments such as GnRH agonists exist, they are indicated for the preoperative short-term use and can be associated with a “flare” effect early during treatment.^2,3^

The Elaris UFs 1 and 2 (UF-1 and UF-2) studies were identical, 6-month, phase 3 randomized trials that evaluated the efficacy and safety of elagolix with add-back therapy in women with fibroid-associated HMB.^4^ In these trials, responders were defined as women who met the primary end point of simultaneously having both menstrual blood loss (MBL) <80 mL and *a* ≥ 50% reduction in MBL from baseline at the final month; nonresponders were defined as women who met neither or only one of the bleeding criteria of the primary end point or women who prematurely discontinued treatment because of adverse events (AEs) or lack of efficacy or required surgical or invasive intervention for UFs, even if they met both bleeding criteria of the primary end point.^4^ As such, the designation “nonresponder” in these trials may erroneously imply that these women did not have any reduction in MBL in response to elagolix with add-back therapy.

Considering the importance of the patient experience in HMB, the purpose of this study was to examine menstrual bleeding outcomes in women who were classified as nonresponders but may have had a clinically meaningful response to elagolix with add-back therapy by meeting one or both of the bleeding criteria of the primary end point.

## Methods

### Study design

This is a *post hoc* analysis of data pooled from two replicate studies Elaris UF-1 and UF-2 (Clinicaltrials.gov identifiers: NCT02654054 and NCT02691494). These two studies were identical in design with the UF-1 study conducted at 76 sites in the United States (including Puerto Rico) from December 2015 through December 2018, and UF-2 was conducted at 77 sites in the United States and Canada from February 2016 through January 2019. One study participant in UF-1 and three participants in UF-2 who underwent randomization were enrolled before the registration date of the trials on Clinicaltrials.gov due to administrative error.

Details of the overall study designs have been published previously.^4^ Briefly, each trial consisted of a washout period of hormonal medications (if applicable), a screening period of 2.5 to 3.5 months, a treatment period of up to 6 months, and a follow-up period of up to 12 months (or a corresponding extension study). At the start of the treatment period, women were randomized (2:1:1) to receive 300 mg of elagolix twice daily with add-back therapy (estradiol 1 mg and norethindrone acetate 0.5 mg once daily), 300 mg of elagolix alone twice daily, or placebo for 6 months. Women who were receiving elagolix alone were included as a reference group to help characterize the impact of add-back therapy on the safety/tolerability and efficacy of elagolix and were not presented in this *post hoc* analysis.

The trials were conducted in accord with the guidelines of the International Council for Harmonisation and applicable regulations and ethical principles of the Declaration of Helsinki. The study protocols were approved by the Schulman Institutional Review Board for central sites and by an institutional review board, ethics committee, or both for all other trial sites. All women provided written informed consent before enrollment.

### Patients and treatments

Eligible participants were premenopausal women aged 18 to 51 years with an ultrasound-confirmed diagnosis of UFs and alkaline hematin-measured HMB, as defined by >80 mL of MBL per menstrual cycle for ≥2 separate cycles.^5^ Women included in the *post hoc* analysis were treated for up to 6 months with elagolix plus add-back therapy or placebo in a matched, double-blind, double-dummy manner.^4^

### Analysis groups

In both trials, nonresponders were defined as women who did not meet the primary end point of simultaneously having both <80 mL MBL and ≥50% MBL reduction from baseline at the final month, or prematurely discontinued treatment because of AEs or lack of efficacy or required surgery or invasive intervention to treat UFs, even if they met both the bleeding criteria of the primary end point.^4^ Responders were defined as women that simultaneously met both primary end point bleeding criteria, with premature discontinuation only allowed due to noncompliance, withdrawal of consent, lost to follow-up, pregnancy, excluding medications, or other prespecified reasons. The final month was defined as the last 28 days before and including the last treatment period visit date (if data on alkaline hematin-measured MBL were available between the last treatment period visit date and the last dose date, then the last dose date was used).^4^

For the current analysis, nonresponders were divided into three groups: (1) women who met both bleeding criteria of the primary end point but prematurely discontinued treatment because of the prespecified reasons; (2) women who met one of the two bleeding criteria, regardless of discontinuation status; and (3) women who met none of the bleeding criteria, regardless of discontinuation status.

### Assessments

Menstrual bleeding outcomes were assessed by least-squares (LS) mean and mean percent change in MBL from baseline to months 1, 3, and 6, which were efficacy end points in the UF-1 and UF-2 trials, and by treatment group and nonresponder classification. The alkaline hematin method was used to objectively measure MBL from used sanitary products collected during the screening and treatment period.^6^ Briefly, the sanitary products were pummeled with sodium hydroxide, which leads to the conversion of hemoglobin to alkaline hematin. The absorbance of alkaline hematin was measured using photometric techniques against calibration curves. By comparing with the woman's serum hemoglobin concentration, the amount of MBL in the sanitary product was determined. Quality of life was assessed with the Uterine Fibroid Symptom and Quality of Life (UFS-QoL) questionnaire at baseline, month 3, month 6, and final month.

Safety was determined by frequency and severity of adverse events (AEs), including standardized Medical Dictionary for Regulatory Activities queries, analyzed by responder status and treatment group.

### Statistical analyses

This analysis was performed in women with observed data and excluded women with missing final month MBL data. Categorical assessments were summarized by frequencies and percentages. LS mean and mean percent (±SE) changes from baseline were obtained from an analysis of covariance model with treatment and study as the main effects and baseline MBL volume as a covariate. For the primary end point bleeding criteria, statistical comparisons between elagolix with add-back therapy group and the placebo group were not performed due to the small sample size of nonresponders in the group receiving elagolix with add-back therapy. For UFS-QoL data, statistical significance was determined using an analysis of covariance model with treatment and study as the main effects and baseline as a covariate. Homogeneity of treatment effect across responder/nonresponder groups for AEs was verified using the Breslow-Day test for any AE reported by ≥10 patients per treatment group within each responder/nonresponder group.

## Results

### Patients

Of the 791 women randomized, a total of 549 women treated with elagolix plus add-back therapy (*n* = 367) or placebo (*n* = 182) with observed final month MBL data in either UF-1 or UF-2 studies were included in the current analysis. Demographics and baseline clinical characteristics are summarized in Table 1. The women were representative of the population of women with symptomatic fibroids. Overall, the mean age was ∼42 years, and 68.3% of women were black or African American. Baseline demographics and disease characteristics—including race, baseline MBL, uterine volume, and fibroid volume—were generally balanced between responders and nonresponders. However, nonresponders in both treatment groups had numerically higher mean MBL and uterine volume.

**Table 1.** Baseline Demographics and Characteristics

Characteristic	Responders		Nonresponders	
	Placebo	Elagolix+add-back therapy	Placebo	Elagolix+add-back therapy
Age (y)	*n* = 16 42.0 ± 5.0	*n* = 278 42.4 ± 5.2	*n* = 166 41.9 ± 5.7	*n* = 89 42.8 ± 5.4
Race	*n* = 16	*n* = 278	*n* = 166	*n* = 89
Black or African American	11 (68.8)	187 (67.5)	115 (69.3)	62 (69.7)
Not black or African American	5 (31.3)	90 (32.5)	51 (30.7)	27 (30.3)
Body mass index (kg/m^2^)	*n* = 16 31.7 ± 6.4	*n* = 277 33.6 ± 6.9	*n* = 166 34.3 ± 7.6	*n* = 89 33.2 ± 7.2
Menstrual blood loss/cycle (mL)	*n* = 16 198.5 ± 93.5	*n* = 278 217.9 ± 134.7	*n* = 166 262.8 ± 180.4	*n* = 89 270.3 ± 173.2
Hemoglobin level (g/dL)	*n* = 16 11.8 ± 1.3	*n* = 278 11.2 ± 1.5	*n* = 166 10.9 ± 1.4	*n* = 89 11.1 ± 1.6
Uterine volume (cm^3^)				
Measured with TAU or TVU	*n* = 16 324.6 ± 203.7	*n* = 278 479.0 ± 369.2	*n* = 166 539.3 ± 425.2	*n* = 89 518.8 ± 439.7
Measured with MRI	*n* = 11 420.6 ± 241.2	*n* = 140 566.5 ± 430.4	*n* = 82 722.5 ± 653.3	*n* = 37 662.5 ± 572.0
Average fibroid volume (cm^3^)				
Measured with TAU or TVU	*n* = 16 30.7 ± 38.8	*n* = 272 51.9 ± 75.5	*n* = 162 65.9 ± 91.5	*n* = 89 64.5 ± 122.8
Measured with MRI	*n* = 11 36.2 ± 39.2	*n* = 132 69.5 ± 71.9	*n* = 76 93.9 ± 113.4	*n* = 35 65.7 ± 60.5

Data are mean ± SD or *n* (%). Add-back therapy defined as estradiol 1 mg/norethindrone acetate 0.5 mg once daily.

SD, standard deviation; TAU, transabdominal ultrasonography; TVU, transvaginal ultrasonography; MRI, magnetic resonance imaging.

#### Responder status

**FIG. 1.**
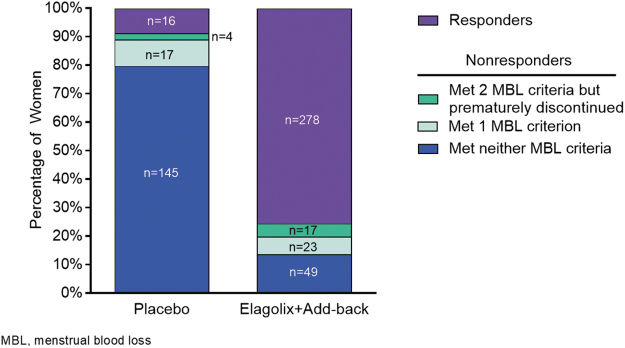
Characteristics of women not categorized as responders to treatment. Percentages of women by treatment and response criteria. Nonresponders were defined as women who did not simultaneously meet both primary end point bleeding criteria (<80 mL MBL and ≥50% reduction from baseline in MBL at final month) or women who simultaneously met both primary end point bleeding criteria (<80 mL MBL and ≥50% reduction from baseline MBL at final month) but prematurely discontinued treatment because of AEs or lack of efficacy or required surgical or invasive fibroid treatment. AEs, adverse events; MBL, menstrual blood loss.

Of the 367 women in the group receiving elagolix with add-back therapy, 278 (76%) were responders who met both bleeding criteria of the primary end point and did not prematurely discontinue treatment for the prespecified reasons, and 89 (24%) met the definition of nonresponder. Of the 89 nonresponders in the group receiving elagolix with add-back therapy, 17 (19%) met both bleeding criteria but prematurely discontinued treatment for the reasons mentioned above, while 23 (26%) met one bleeding criterion and 49 (55%) met neither bleeding criteria, regardless of discontinuation status (Fig. 1). Of the 23 women who met just one of the bleeding criteria, most women achieved *a* ≥ 50% reduction from baseline (21 [91.3%]) rather than <80 mL MBL in the final month (2 [8.7%]).

As expected, of the 166 nonresponders in the placebo group, the majority were classified as such because they did not meet either of the bleeding criteria of the primary end point (*n* = 145, 87.3%); only 4 (2.4%) respondents met both bleeding criteria but prematurely discontinued and 17 (10.2%) met one of the bleeding criteria, regardless of premature discontinuation status. Of the 17 patients who met one criterion, 10 (58.8%) achieved <80 mL in the final month and seven (41.2%) achieved *a* ≥ 50% reduction in MBL from baseline.

### Efficacy

LS mean absolute and percent changes from baseline in MBL are summarized in Table 2. Of the 17 nonresponders in the group receiving elagolix with add-back therapy who met both bleeding criteria but prematurely discontinued treatment for the prespecified reasons, LS mean changes in MBL from baseline were −183.1 ± 23.5 mL (confidence interval [95% CI] −231.6 to −134.6, *n* = 13) at month 1 and −280.8 ± 6.6 mL (95% CI −297.8 to −263.8, *n* = 6) at month 3. Furthermore, their LS mean percent changes in MBL from baseline were −80.3% ± 9.6% (95% CI −100.1 to −60.6, *n* = 13) at month 1 and −86.2% ± 5.1% (95% CI −99.3 to −73.1, *n* = 6) at month 3, revealing that, on average, this group of women had *a* ≥ 50% reduction in MBL from baseline as early as month 1, which persisted through month 3. These 17 women had no data available at month 6.

**Table 2.** Mean Absolute and Percent Change From Baseline in Menstrual Blood Loss Over Time by Treatment Group and Nonresponder Classification

	Met both MBL criteria but discontinued prematurely		Met 1 MBL criterion		Met neither MBL criterion		All nonresponders	
	Placebo	Elagolix+add-back therapy	Placebo	Elagolix+add-back therapy	Placebo	Elagolix +add-back therapy	Placebo	Elagolix+add-back therapy
Baseline	*n* = 4	*n* = 17	*n* = 17	*n* = 23	*n* = 145	*n* = 49	*n* = 166	*n* = 89
Mean (mL)	227.1 ± 161.4	238.8 ± 176.8	258.2 ± 183.4	328.9 ± 173.8	264.3 ± 181.6	253.7 ± 168.6	262.8 ± 180.4	270.3 ± 173.2
Change from baseline								
1 month	*n* = 3	*n* = 13	*n* = 17	*n* = 21	*n* = 141	*n* = 45	*n* = 161	*n* = 79
Change (mL)	–66.3 ± 50.4	–183.1 ± 23.5	–95.3 ± 38.3	–59.0 ± 34.3	–11.0 ± 14.4	–49.7 ± 25.8	–19.1 ± 13.3	–79.5 ± 19.0
Change (%)	–39.2 ± 20.5	–80.3 ± 9.6	–25.1 ± 10.8	–16.1 ± 9.7	–5.6 ± 4.8	–14.8 ± 8.7	–8.0 ± 4.4	–26.2 ± 6.2
3 months	—	*n* = 6	*n* = 16	*n* = 18	*n* = 128	*n* = 33	*n* = 144	*n* = 57
Change (mL)		–280.8 ± 6.6	–80.7 ± 30.7	–210.0 ± 28.6	–0.8 ± 13.1	–90.8 ± 26.0	–7.1 ± 12.9	–157.8 ± 20.7
Change (%)		–86.2 ± 5.1	–28.7 ± 9.4	–72.9 ± 8.7	–2.8 ± 4.3	–41.3 ± 8.6	–5.5 ± 4.0	–57.0 ± 6.3
6 months	—	—	*n* = 13	*n* = 16	*n* = 110	*n* = 19	*n* = 123	*n* = 35
Change (mL)			–138.4 ± 17.8	–180.5 ± 16.1	24.4 ± 17.2	12.0 ± 41.4	9.4 ± 16.0	–77.0 ± 30.2
Change (%)			–43.9 ± 6.9	–62.7 ± 6.3	13.0 ± 6.2	1.1 ± 14.9	7.2 ± 5.7	–27.2 ± 10.7

Baseline values are expressed as mean ± SD. Changes from baseline are presented as LS mean ± SE or mean percent ± SE obtained from an analysis of covariance model with treatment and study as the main effects and baseline MBL volume as a covariate.

LS, least-squares; MBL, menstrual blood loss.

Among the 23 nonresponders in the group receiving elagolix with add-back therapy who met one of the two bleeding criteria, regardless of discontinuation status, LS mean changes in MBL from baseline were −59.0 ± 34.3 mL (95% CI −128.5 to 10.5, *n* = 21) at month 1, −210.0 ± 28.6 mL (95% CI −268.1 to −151.9, *n* = 18) at month 3, and −180.5 ± 16.1 mL (95% CI −213.6 to −147.4, *n* = 16) at month 6. Their LS mean percent changes in MBL from baseline were −16.1% ± 9.7% (95% CI −35.7 to 3.5, *n* = 21) at month 1, −72.9% ± 8.7% (95% CI −90.6 to −55.1, *n* = 18) at month 3, and −62.7% ± 6.3% (95% CI −75.5 to −49.8, *n* = 16) at month 6, revealing that this group of women, on average, had *a* ≥ 50% reduction in MBL at months 3 and 6 and a numerical trend of improvement from baseline in MBL over time. Mean reductions in MBL in this group of women treated with elagolix and add-back therapy were numerically greater than in the placebo group starting at month 3 and through month 6.

Among nonresponders who met none of the bleeding criteria of the primary end point, women receiving elagolix with add-back therapy also achieved a numerically greater mean change in MBL than did those in the placebo group at month 1 (−49.7 ± 25.8 mL vs. −11.0 ± 14.4 mL) and month 3 (−90.8 ± 26.0 mL vs. −0.8 ± 13.0 mL); however, both groups showed increases in MBL compared with baseline at month 6 (12.0 ± 41.4 and 24.4 ± 17.2 mL in the group receiving elagolix with add-back therapy and placebo groups, respectively).

Among nonresponders, women treated with elagolix+add-back also demonstrated improvements in quality of life (Table 3). The mean improvement in UFS-QoL Health-Related Quality of Life total score was significantly greater than placebo at 3 months (13.2 ± 1.8 vs. 25.5 ± 2.9, *p* < 0.001) and final month of treatment (8.4 ± 1.8 vs. 15.9 ± 2.8, *p* = 0.025), with numerically greater improvements observed at 6 months (8.6 ± 1.9 vs. 16.2 ± 3.5, *p* = 0.054), perhaps due to the smaller number of women at this timepoint. Changes in UFS-QoL Symptom Severity scores among the elagolix+add-back nonresponders were significantly improved versus placebo at 3 months (−16.3 ± 1.7 vs. −24.4 ± 2.7, *p* < 0.01), with a similar, but not statistically significant, trend at final month of treatment (−8.9 ± 1.7 vs. −13.5 ± 2.6, *p* = 0.09, Table 3).

**Table 3.** Uterine Fibroid Symptom and Quality of Life Questionnaire Changes for All Women Categorized as Nonresponders

	Placebo	Elagolix+add-back therapy
Symptom severity		
Baseline Mean ± SE	*n* = 150 60.4 ± 1.7	*n* = 66 58.8 ± 2.6
3 months Change from baseline *p*	*n* = 146 −16.3 ± 1.7	*n* = 60 −24.4 ± 2.7 0.01^**^
6 months Change from baseline *p*	*n* = 135 −8.9 ± 1.7	*n* = 39 −11.2 ± 3.2 0.52
Final month Change from baseline *p*	*n* = 150 −8.3 ± 1.7	*n* = 66 −13.5 ± 2.6 0.09
HRQoL Total		
Baseline Mean ± SE	*n* = 149 43.1 ± 1.8	*n* = 66 46.6 ± 2.8
3 months Change from baseline *p*	*n* = 145 13.2 ± 1.8	*n* = 60 25.5 ± 2.9 <0.001^***^
6 months Change from baseline *p*	*n* = 134 8.6 ± 1.9	*n* = 39 16.2 ± 3.5 0.054
Final month Change from baseline *p*	*n* = 149 8.4 ± 1.8	*n* = 66 15.9 ± 2.8 0.025^*^

Unless otherwise noted, values are LS mean ± standard error obtained from an analysis of covariance model with treatment and study as the main effects and baseline as a covariate. Symptom Severity scores range from 0 to 100 with higher scores indicating increased severity. HRQoL scores range from 0 to 100 with higher scores indicating better quality of life.

HRQoL, health-related quality of life; SE, standard error.

### Safety

Results of the safety analyses are summarized in Table 4. Rates of overall AEs were similar between treatment groups, regardless of response status, and ranged from 67.5% in nonresponders to placebo to 76.4% in nonresponders to elagolix. No major differences were observed between groups or by responder status in severe or serious AEs. The rate of AEs leading to treatment discontinuation was generally higher among nonresponders to elagolix (29.2%) than nonresponders to placebo (7.2%), responders to placebo (6.3%), or responders to elagolix (0.4%). Similar to previously published results for UF-1 and UF-2,^4^ the most common AEs for responders and nonresponders to elagolix, respectively, were hot flush (20.1% and 20.2%), nausea (7.6% and 13.5%), headache (7.9% and 12.4%), fatigue (6.1% and 7.9%), and night sweats (9.0% and 6.7%).^4^ Of the 17 patients who met both primary end point criteria but discontinued prematurely, AEs leading to discontinuation in more than one patient include headaches (*n* = 3), hot flushes (*n* = 2), nausea (*n* = 2), and lower abdominal pain (*n* = 2). These were consistent with the most common AEs reported in the overall study population.

**Table 4.** Summary of Adverse Events

Characteristic	Responders		Nonresponders	
	Placebo (*n* = 16)	Elagolix+add-back therapy (*n* = 278)	Placebo (*n* = 166)	Elagolix+add-back therapy (*n* = 89)
All AEs	12 (75.0)	193 (69.4)	112 (67.5)	68 (76.4)
Severe AE	2 (12.5)	23 (8.3)	8 (4.8)	10 (11.2)
Serious AE	0	9 (3.2)	5 (3.0)	0
AE leading to treatment discontinuation	1 (6.3)	1 (0.4)	12 (7.2)	26 (29.2)
Most common AEs with elagolix+add-back therapy				
Hot flush	3 (18.8)	56 (20.1)	10 (6.0)	18 (20.2)
Nausea	1 (6.3)	21 (7.6)	18 (10.8)	12 (13.5)
Headache	0	22 (7.9)	13 (7.8)	11 (12.4)
Fatigue	0	17 (6.1)	6 (3.6)	7 (7.9)
Night sweats	1 (6.3)	25 (9.0)	7 (4.2)	6 (6.7)

Data are *n* (%).

AE, adverse event.

## Discussion

Guidance from the American College of Obstetricians and Gynecologists (ACOG) acknowledges that, although a criterion of >80 mL MBL is used to define HMB for clinical research, diagnosis of HMB in clinical practice should be based on patient perception.^7^ This patient-centric assessment of HMB was supported more recently by the National Institute for Health and Care Excellence (NICE) that presented an updated definition of HMB as “excessive menstrual blood loss which interferes with a woman's physical, social, emotional and/or material quality of life.”^8^

The UF-1 and UF-2 trials used the standard clinical research definition of HMB as >80 mL MBL per cycle, as measured by the alkaline hematin method, in addition to the change criterion of ≥50% reduction in MBL from baseline to the final month. In clinical practice, MBL is not typically measured; rather, HMB is diagnosed based on the patient's perception of excessive bleeding, in agreement with the definitions by ACOG and NICE.^7,8^ Evidence also supports a perception-based definition and suggests that a volumetric criterion of >80 mL MBL is not ideal for clinical use, as some women perceive MBL >80 mL as manageable, while others consider MBL <80 mL intolerable.^9^ For example, in an investigation of 952 women who were referred to a gynecologic clinic for heavy periods, the majority (66%) had a median MBL of 53 mL per cycle (well under the 80 mL clinical research standard).^10^ There is great variation in baseline MBL among patients, and a volumetric target alone may be of limited utility in determining clinical benefit. For some patients, a change in MBL may be more meaningful than achieving a specific volumetric target.

In the UF-1 and UF-2 clinical trials, a total of 89 of 376 (24%) women with fibroid-related HMB treated with elagolix plus add-back therapy were considered nonresponders based on the definition of the primary end point, yet this *post hoc* analysis demonstrates that most of these women had some improvement which could be clinically meaningful and challenges the notion that “nonresponders” achieve no benefit. Of the 89 patients considered nonresponders, 40 (45%) may have experienced a clinically meaningful response to elagolix with add-back therapy, as 23 of these patients met one of the two bleeding criteria, regardless of discontinuation status, nearly all (91.3%) of which met the ≥50% reduction from baseline. The remaining 17 patients achieved both bleeding criteria and would have been considered responders had they not prematurely discontinued treatment. Although nearly all (*n* = 16) of these women discontinued prematurely because of AEs, it is important to note that there were no important differences in overall AEs between responders and nonresponders. Moreover, treatment with elagolix+add-back significantly improved the quality of life in the nonresponder group. In summary, these results indicate that nearly half of patients designated as nonresponders met either one or both of the primary end point bleeding criteria, indicating reduced HMB over time, and displayed improvements in quality of life.

These findings raise several important questions for future research, especially with the recent approval of elagolix with add-back therapy for the management of HMB associated with UFs and the anticipated increased use in clinical practice. First, it will be important to better understand why women who achieve improved MBL (suggesting clinical efficacy) with treatment would discontinue treatment. In addition, we need a better understanding of the variables underlying the perception of improvement in MBL and how this relates to quantitative changes in MBL or achievement of specific volumetric goals. This knowledge, in addition to understanding if certain patient baseline characteristics may predict clinical benefit with elagolix for women with HMB due to UFs, will help women and their providers make appropriate clinical decisions.^11^

Strengths of this study include the use of a large, diverse patient population from two phase 3, double-blind, randomized clinical trials with patients from the United States and Canada and the fact that the study population is representative of the typical group of women most impacted by fibroids. Moreover, we report a quantitative assessment of bleeding outcomes. Limitations include the use of *post hoc* analysis and low participant numbers in some groups, limiting the power of comparisons for this analysis.

## Conclusion

Taken together, the results of this pooled *post hoc* analysis suggest that nearly half of patients taking elagolix plus add-back therapy who were considered nonresponders in the 2 phase 3 clinical trials had reductions in MBL that may be clinically significant to both patients and physicians. Considering the patient-centered approach to diagnosis and resolution of HMB, the results of this nonresponder analysis support further consideration of this medical treatment option for women with HMB.

## Data Sharing Statement

AbbVie is committed to responsible data sharing regarding the clinical trials we sponsor. This includes access to anonymized, individual, and trial-level data (analysis data sets), as well as other information (*e.g.*, protocols and Clinical Study Reports), as long as the trials are not part of an ongoing or planned regulatory submission. These clinical trial data can be requested by any qualified researchers who engage in rigorous, independent scientific research and will be provided following review and approval of a research proposal and Statistical Analysis Plan (SAP) and execution of a Data Sharing Agreement (DSA). Data requests can be submitted at any time, and the data will be accessible for 12 months, with possible extensions considered. For more information on the process, or to submit a request, visit the following link: https://www.abbvie.com/our-science/clinical-trials/clinical-trials-data-and-information-sharing/data-and-information-sharing-with-qualified-researchers.html

## References

[1] Bulun SE. Uterine Fibroids. N. Engl. J. Med 2013;369:1344–1355. 10.1056/NEJMra1209993 24088094

[2] U.S. Food and Drug Administration website. LUPRON DEPOT (leuprolide acetate for depot suspension 3.75 mg [package insert]. North Chicago, IL, USA: AbbVie Inc.; 2013. Available at: https://www.accessdata.fda.gov/drugsatfda_docs/label/2012/019943s031s,020708s031lbl.pdf, Accessed March 28, 2021.

[3] Knobil E. Remembrance: The discovery of the hypothalamic gonadotropin-releasing hormone pulse generator and of its physiological significance. Endocrinology 1992;131:1005–1006. 1505445 10.1210/endo.131.3.1505445

[4] Schlaff WD, Ackerman RT, Al-Hendy A, et al. Elagolix for heavy menstrual bleeding in women with uterine fibroids. N Engl J Med 2020;382:328–340. 31971678 10.1056/NEJMoa1904351

[5] Duckitt K, Collins S. Menorrhagia. BMJ Clin Evid 2008;2008:0805. PMC2907973 19445802

[6] Magnay JL, O'Brien S, Gerlinger C, Seitz C. A systematic review of methods to measure menstrual blood loss. BMC Womens Health 2018;18:142. 30134884 10.1186/s12905-018-0627-8 PMC6106944

[7] American College of Obstetricians and Gynecoloists. Diagnosis of abnormal uterine bleeding in reproductiveaged women. Obstet Gynecol 2012;120:197–206. 22914421 10.1097/AOG.0b013e318262e320

[8] National Institute for Health and Care Excellence: Heavy menstrual bleeding: assessment and management. NICE guideline [NG88]. 2020. Available at: https://www.nice.org.uk/guidance/ng88/resources/heavy-menstrual-bleeding-assessment-and-management-pdf-1837701412549 Accessed May 30, 2020. 34101395

[9] Warner PE, Critchley HO, Lumsden MA, et al. Menorrhagia II: Is the 80-mL blood loss criterion useful in management of complaint of menorrhagia? Am J Obstet Gynecol 2004;190:1224–1229. 15167822 10.1016/j.ajog.2003.11.016

[10] Warner PE, Critchley HO, Lumsden MA, et al. Menorrhagia I: Measured blood loss, clinical features, and outcome in women with heavy periods: A survey with follow-up data. Am J Obstet Gynecol 2004;190:1216–1223. 15167821 10.1016/j.ajog.2003.11.015

[11] Al-Hendy A, Bradley L, Owens CD, et al. Predictors of response for elagolix with add-back therapy in women with heavy menstrual bleeding associated with uterine fibroids. Am J Obstet Gynecol 2020;224:72.e1–e72.e50. 32702363 10.1016/j.ajog.2020.07.032 PMC8800453

